# A model for stimulation of enzyme activity by a competitive inhibitor based on the interaction of terazosin and phosphoglycerate kinase 1

**DOI:** 10.1073/pnas.2318956121

**Published:** 2024-02-20

**Authors:** Mitchell J. Riley, Colleen C. Mitchell, Sarah E. Ernst, Eric B. Taylor, Michael J. Welsh

**Affiliations:** ^a^Department of Mathematics, University of Iowa, Iowa City, IA 52242; ^b^Department of Internal Medicine, Pappajohn Biomedical Institute, Roy J. and Lucille A. Carver College of Medicine, University of Iowa, Iowa City, IA 52242; ^c^HHMI, University of Iowa, Iowa City, IA 52242; ^d^Department of Molecular Physiology and Biophysics, Pappajohn Biomedical Institute, Roy J. and Lucille A. Carver College of Medicine, University of Iowa, Iowa City, IA 52242

**Keywords:** enzyme, metabolism, Parkinson’s disease, terazosin, phosphoglycerate kinase 1

## Abstract

The drug terazosin (TZ) binds to and can enhance the activity of the glycolytic enzyme phosphoglycerate kinase 1 (PGK1) and can increase ATP levels. That finding prompted studies of TZ in Parkinson’s disease (PD) in which decreased neuronal energy metabolism is a hallmark feature. TZ was neuroprotective in cell-based and animal PD models and in large epidemiological studies of humans. However, how TZ might increase PGK1 activity has remained a perplexing question because structural data revealed that the site of TZ binding to PGK1 overlaps with the site of substrate binding, predicting that TZ would competitively inhibit activity. Functional data also indicate that TZ is a competitive inhibitor. To explore the paradoxical observation of a competitive inhibitor increasing enzyme activity under some conditions, we developed a mass action model of TZ and PGK1 interactions using published data on PGK1 kinetics and the effect of varying TZ concentrations. The model indicated that TZ-binding introduces a bypass pathway that accelerates product release. At low concentrations, TZ binding circumvents slow product release and increases the rate of enzymatic phosphotransfer. However, at high concentrations, TZ inhibits PGK1 activity. The model explains stimulation of enzyme activity by a competitive inhibitor and the biphasic dose–response relationship for TZ and PGK1 activity. By providing a plausible mechanism for interactions between TZ and PGK1, these findings may aid development of TZ or other agents as potential therapeutics for neurodegenerative diseases. The results may also have implications for agents that interact with the active site of other enzymes.

## Significance

Terazosin (TZ) can enhance energy metabolism and slow disease progression in models of neurodegenerative diseases. TZ increases the activity of phosphoglycerate kinase 1 (PGK1), the first adenosine triphosphate (ATP) generating step in glycolysis, and increases cellular ATP levels. However, TZ competitively inhibits PGK1, and thus, it is perplexing how it might increase PGK1 activity. A mass action model of the TZ:PGK1 interaction reveals that at low concentrations, TZ-binding introduces a bypass pathway that circumvents slow product release from PGK1 and accelerates enzymatic activity. This plausible mechanism may aid development of therapeutics for neurodegenerative diseases and may explain unexpected results with agents that interact with the active site of other enzymes.

Neurodegenerative diseases pose an enormous personal and public health challenge, and the challenge is increasing as the population ages. However, we lack treatments to slow or prevent the progressive neuron destruction in Parkinson’s disease (PD), Alzheimer’s disease, Huntington’s disease, and amyotrophic lateral sclerosis. A common feature of these diseases is impaired energy metabolism (1–4). Previous studies showed that terazosin (TZ), an FDA-approved drug developed to treat hypertension and benign prostatic hyperplasia, has an additional target, phosphoglycerate kinase 1 (PGK1), the first ATP-generating enzyme in glycolysis (5).

The glycolytic enzyme PGK1 catalyzes reversible phosphotransfer from 1,3-bisphosphoglycerate (1,3-BPG) to adenosine diphosphate (ADP), producing 3-phosphoglycerate (3-PG) and ATP (6). By stimulating PGK1 activity, TZ can enhance energy metabolism in two ways; PGK1 produces ATP itself, and 3-PG serves as a glycolytic substrate leading to increased oxidative phosphorylation and ATP production (5, 7). Previous work showed that TZ can increase cellular ATP levels in cells and animals, and it prevented cell death in PD and other models of neurodegenerative disease (7–12). Moreover, multiple investigations of large human databases indicate that the use of TZ is associated with reduced symptoms and delayed onset of PD (7, 13–16).

However, a mechanism by which TZ might activate PGK1 has been perplexing. The crystal structure of PGK1 and TZ showed that TZ binds to PGK1 at its ADP/ATP binding pocket with the quinazoline portion of TZ overlapping with the adenine ring of ADP (5). That location predicts that TZ would inhibit PGK1 activity by preventing ADP binding, likely as a competitive inhibitor. Consistent with that prediction, high concentrations of TZ inhibited the activity of isolated PGK1 protein and decreased ATP production in cultured cells (7). The relationship between TZ concentrations and activity of PGK1 has also been puzzling; low concentrations of TZ produce a small stimulation of PGK1 activity, and high concentrations inhibit PGK1 activity. This unusual biphasic activity pattern was also observed in ATP levels both in cultured cells and in animal models (5, 7).

The primary goal of this study was to address the paradox of how a competitive inhibitor might stimulate PGK1 enzymatic activity. To explore this problem, we developed a mathematical mass action model of the interactions of TZ with PGK1. We began by considering all the forms of PGK1 alone, bound to substrates, products, and TZ. We then considered the links between all forms, and to those links we applied kinetic parameters from earlier studies (5, 6). We calculated the initial rates of product formation at quasi-equilibrium conditions, which would be established before significant conversion of substrate to product. The model suggests that TZ introduces a bypass pathway that accelerates or inhibits PGK1 activity depending on the TZ concentration.

## Model Development

**Fig. 1.**
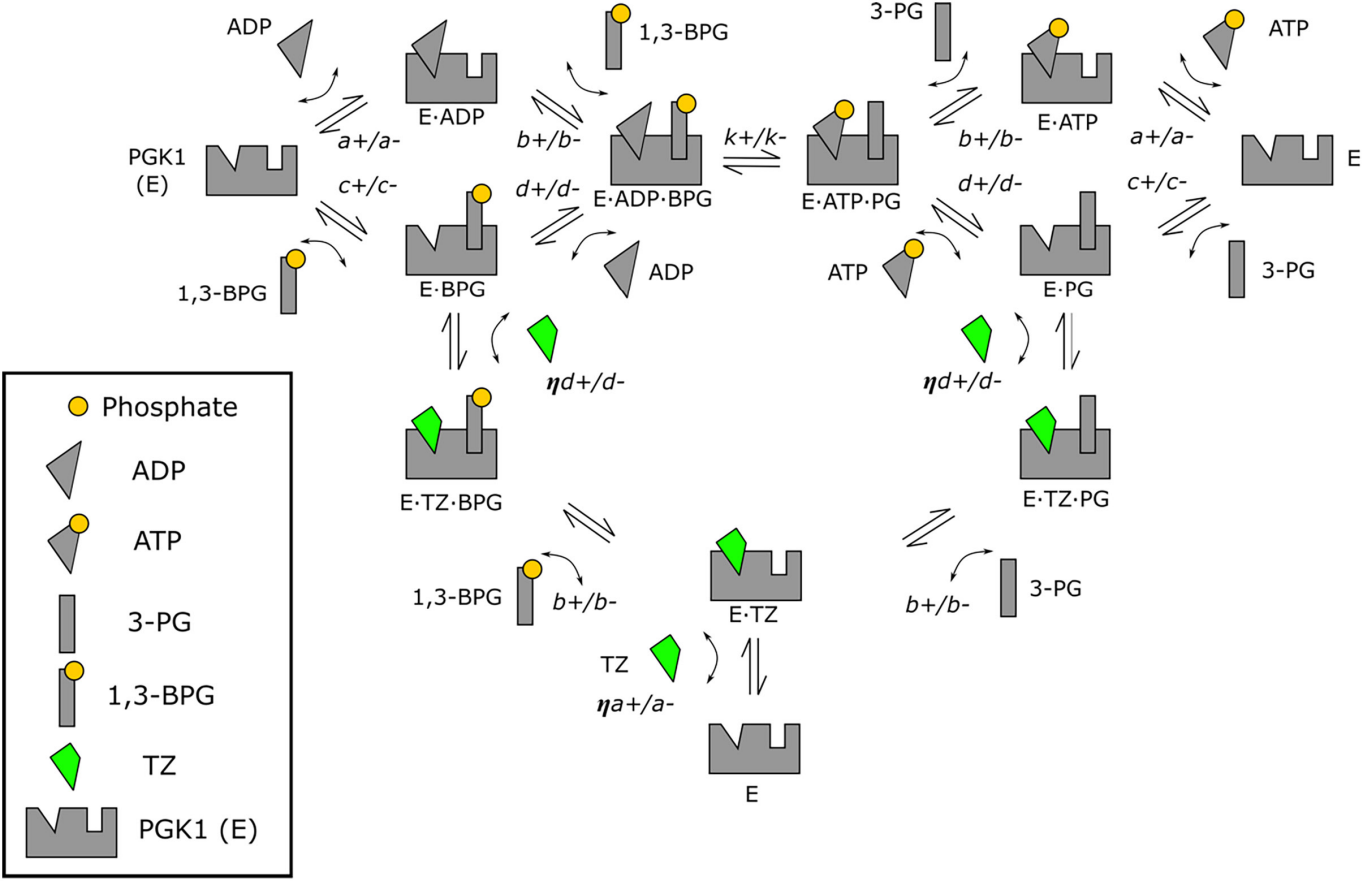
A mass action model describes the interactions of PGK1 with substrates, products, and TZ. A compartmental diagram of each configuration and rate parameter for the mathematical model. The parallel arrows indicate that each interaction is reversible. The *Upper Left* diamond refers to PGK1’s interaction with ADP and 1,3-BPG, and the *Upper Right* diamond refers to PGK1’s interaction with ATP and 3-PG. TZ (green) competes with ADP and ATP for their respective binding pocket on PGK1. A fully developed mathematical model is provided in *SI Appendix*.

The model with all PGK1 forms and the interactions between all forms is shown in Fig. 1. For simplicity and clarity, we refer to PGK1 as “E” and bound PGK1 as “E⋅molecule”, e.g., PGK1 bound to ADP is “E⋅ADP”. Moreover, each substrate or product binds PGK1 randomly based on data for PGK1 (6).

The kinetic rate parameters are the bimolecular association and dissociation rate constants determined by Lallemand et al. (Table 1). The parameters *a_±_, b_±_, c_±_*, and *d_±_* correspond to the association/dissociation of ADP and 1,3-BPG to the various PGK1 forms (E, E⋅ADP, E⋅BPG_,_ and E⋅ADP⋅BPG in the *Top Left* diamond of Fig. 1). Because the association parameters for ATP and 3-PG have not been experimentally determined, we used the corresponding parameters for ADP and 1,3-BPG (Table 1 and Fig. 1). For example, we set parameter *c_±_* (Table 1), which describes 1,3-BPG association or dissociation to E and E⋅BPG to also describe association or dissociation of 3-PG to E and E⋅PG. Likewise, we set parameter *b_±_* (Table 1), which describes 1,3-BPG association or dissociation to E⋅ADP and E⋅ADP⋅BPG to also describe association or dissociation of 3-PG to E⋅ATP and E⋅ATP⋅PG and to E⋅TZ and E⋅TZ⋅PG. In a similar fashion, *a_+_* and *d_+_* are applied as shown in Fig. 1 and Table 1. The unknown phosphotransfer parameters *k_+_* and *k_−_* are both assigned the value 5.0 s^−1^. Finally, the unitless parameter *η* is TZ’s association parameter factor. Compared to ADP and ATP, TZ has greater binding to PGK1 as indicated by a dissociation constant (*Kd*) for PGK1 that is ~10 times lower than ADP and ~100 times lower than ATP (5, 17). In the simulations, we used *η* = 562 to qualitatively match the biphasic response at the same TZ concentrations as previously reported (5). A complete formulation of the differential equations is in *SI Appendix*.

**Table 1.** Kinetic rate parameters for PGK1.

Parameter	Association (+) (s^−1^ µM^−1^)	Dissociation (−) (s^−1^)
*a*	6.1 ± 0.3	38 ± 10
*b*	170 ± 30	160 ± 30
*c*	450 ± 40	14 ± 26
*d*	4.1 ± 0.5	270 ± 30

^*^Parameters *a±*, *b±*, *c±*, and *d±* are shown as the mean with SE from ref. 6. These parameters were determined for the substrates, but as described in the text, we also used these for product formation and release.

For the conditions, we simulated 1,3-BPG production from the Chen et al experiment (5). We fixed 1,3-BPG at 80 μM, which is extrapolated from data from ref. 18. The remaining initial conditions were those described for the in vitro experiments of Chen et al. (5): 0.04 μM PGK1, 1 mM ADP, 0 μM 3-PG, 0 μM ATP, and TZ concentrations of 0, 2.5 nM, 25 nM, 50 nM, 0.25 μM, 0.5 μM, 2.5 μM, and 25 μM. We calculated product formation under quasi-equilibrium conditions at 1 min, a time at which the product production rate has reached and is still at maximum. All simulations were performed in MATLAB.

We define net flux between enzyme forms as the difference between the forward and reverse rates. As an example, *a_+_*[E][ADP] is the forward rate, and *a-*[E⋅ADP] is the reverse rate in the *Upper Left* reaction of Fig. 1. Thus, the net flux of the interaction of PGK1 with ADP is *a_+_*[E][ADP] *-a-*[E⋅ADP].

## Results

**Fig. 2.**
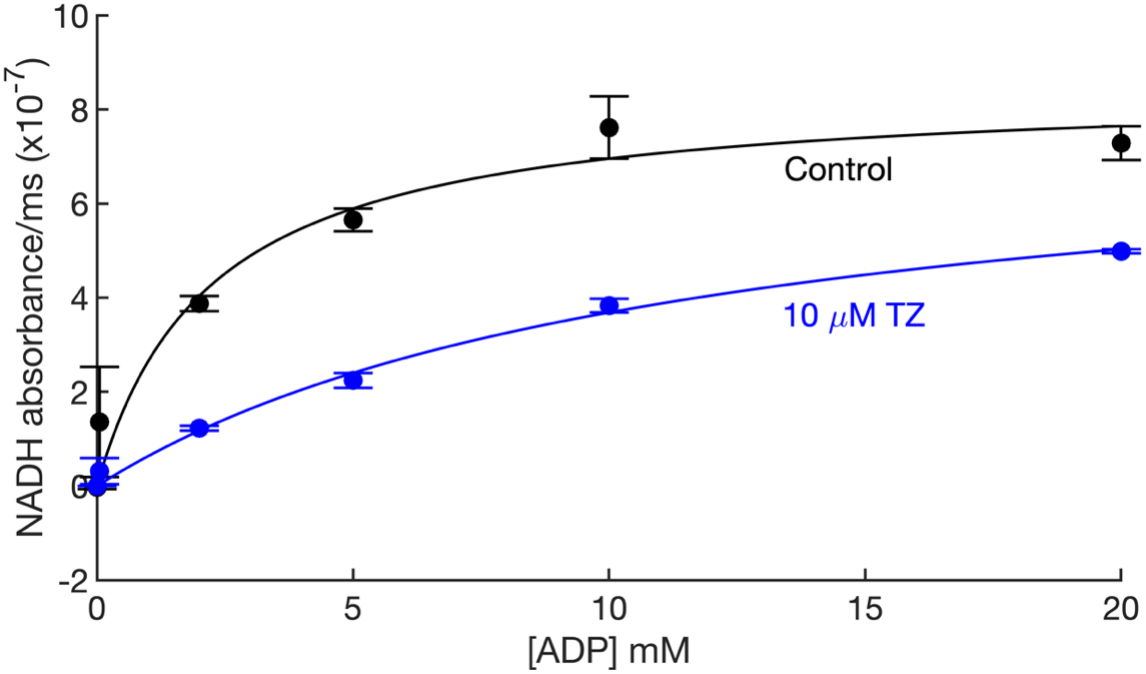
TZ competitively inhibits PGK1 activity. The data are nicotinamide adenine dinucleotide (NADH) absorbance (a measure of PGK1 activity) at increasing concentrations of ADP without and with 10 µM TZ. The curves are the Michaelis–Menten fit to the data. The estimated *K_d_*^’^ for ADP is 2.2 ± 1.0 mM in the absence and 11.5 ± 2.3 mM in the presence of TZ 10 μM (mean ± SD). The estimated *V_max_* is 8.5 × 10^−7^ ± 9.2 × 10^−8^ NADH absorbance/ms in the absence and 7.9 × 10^−7^ ± 7.6 × 10^−8^ NADH absorbance/ms in the presence of TZ 10 μM (mean ± SD).

To further assess the effect of TZ as a competitive inhibitor of PGK1, we tested ADP-dependent PGK1 activity in the absence and presence of 10 μM TZ (Fig. 2). TZ increased the apparent *K*_m_ for ADP from 2.2 mM to 11.5 mM, consistent with competitive inhibition (19).

**Fig. 3.**
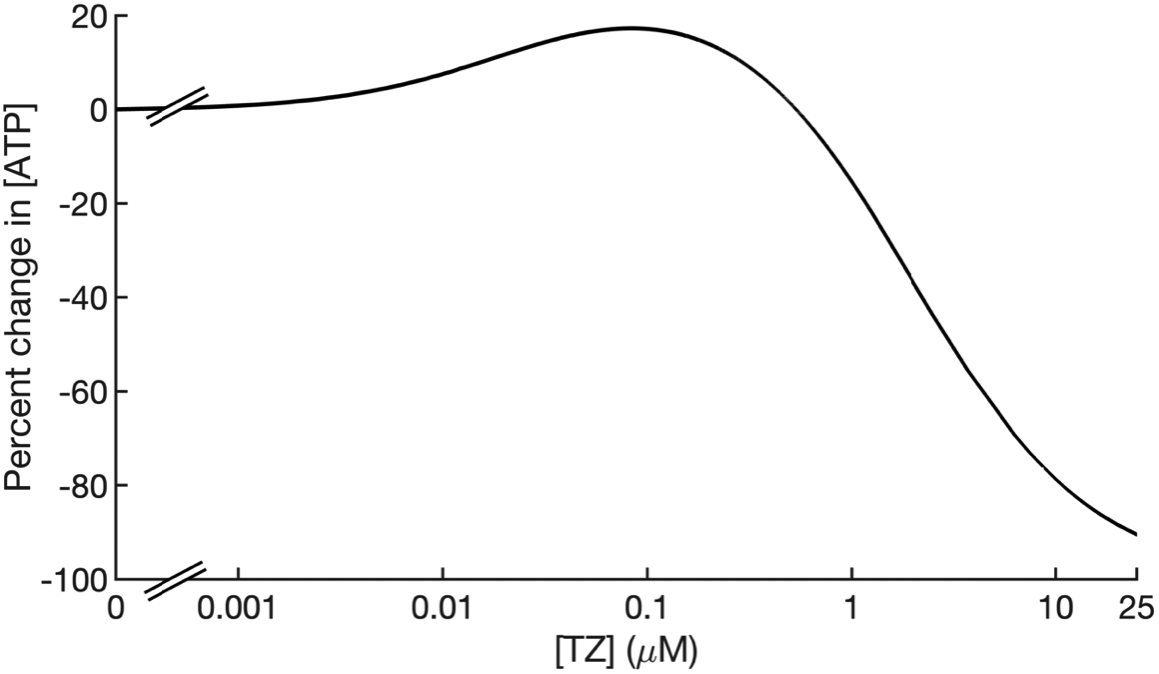
The concentration of TZ alters ATP production. A simulation showing the percent change in ATP production at varying TZ concentrations at 1 min using parameters from Table 1 and initial conditions outlined in the methods.

The mass action model showed that as the TZ concentration increased, TZ stimulated PGK1 activity and ATP production increased, and at higher concentrations of TZ, ATP production fell (Fig. 3).

**Fig. 4.**
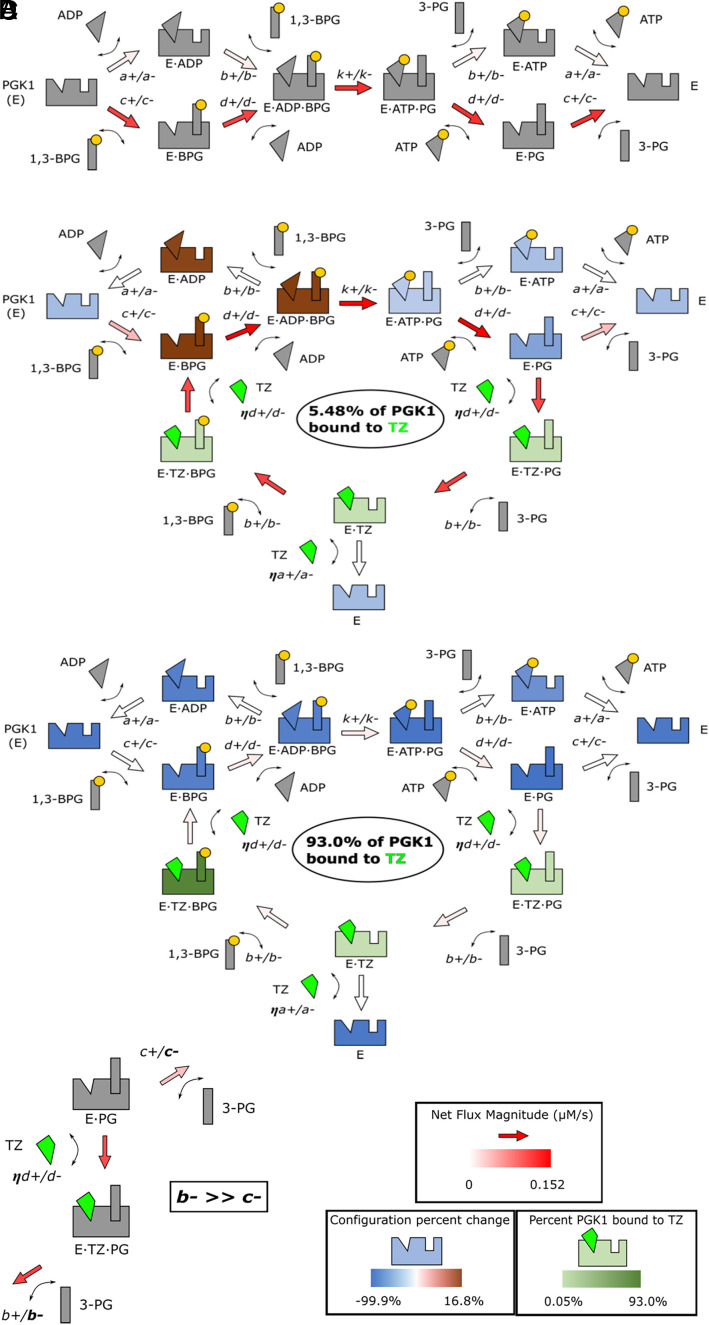
The mass action model predicts the interactions of PGK1, substrates, products, and TZ. (*A*) The PGK1 reaction with no TZ. Arrows indicate the preference for association or dissociation at an interaction. The shade of red indicates the magnitude of the flux for each interaction (refer to Table 2). Configurations are gray because their concentrations were used to calculate the percent changes for non-zero TZ concentrations in panels (*B*–*D*). (*B*) PGK1 effect of 50 nM TZ on interactions. Non-TZ configurations are colored as indicated (Table 2). (*C*) Effect of 25 µM TZ on PGK1 interactions. (*D*) The release of 3-PG from E⋅PG and E⋅TZ⋅PG (expanded portion of panel *B*).

It seems paradoxical that a competitive inhibitor would stimulate enzymatic activity. Therefore, to gain further insight into the potential mechanism, we calculated the effect of TZ concentration on net fluxes for each interaction between enzyme configurations (Table 2). We also calculated the percentage of PGK1 bound to TZ and the percentage change in each enzyme configuration not bound to TZ (Table 3). To facilitate understanding of the numerical data, we graphed the net fluxes and percentage changes without TZ, with 50 nM TZ (which predicted the greatest increase in ATP, ~17%), and with 25 μM TZ (which produced the largest decrease in ATP, ~90%) (Fig. 4 *A*–*C*).

**Table 2.** Calculations for net fluxes with varying TZ concentrations

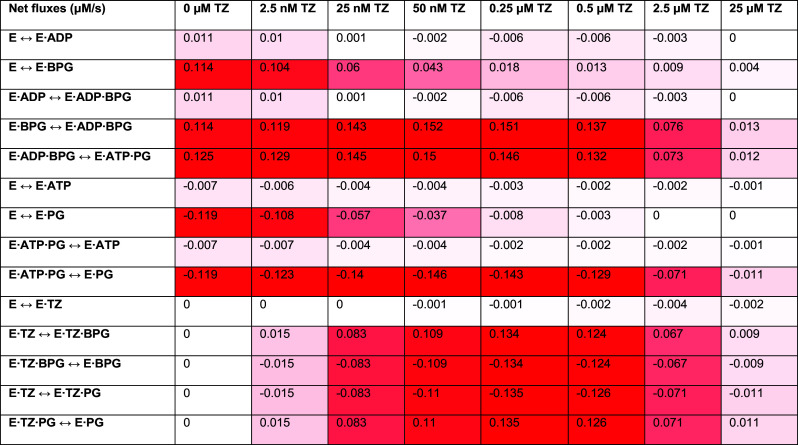

^*^Net fluxes for each interaction. The first column shows each interaction. Additional columns show the net flux between each configuration at the indicated TZ concentrations. Shades of red indicate the magnitude of the net flux with darker shades indicating values further from zero. White net flux indicates a value close to zero.

**Table 3.** Changes in configurations induced by TZ

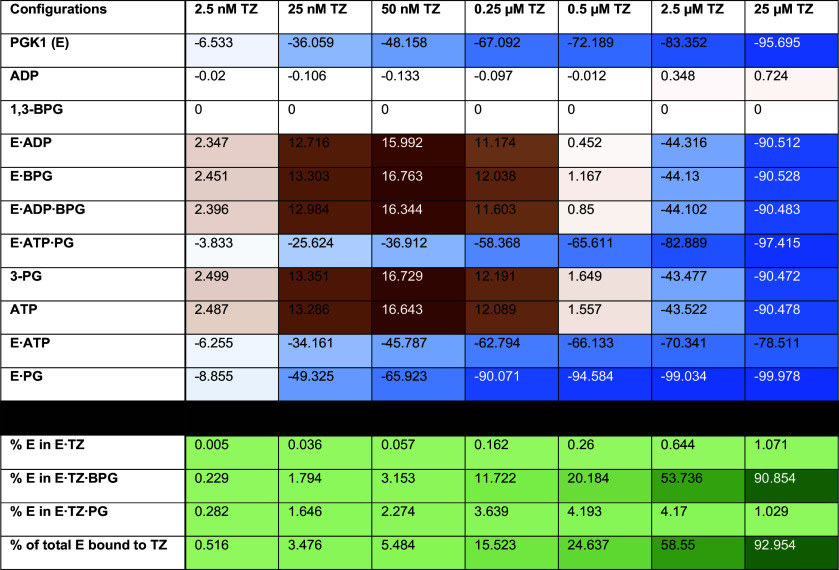

^*^Percent change of each non-TZ configuration (top rows) and the calculated percent of PGK1 bound to TZ (bottom rows). For top rows, brown indicates an increase, blue indicates a decrease, and white indicates no change. For bottom rows, percent change for TZ configurations cannot be calculated because the zero concentration is the baseline. Instead, values are the percent of PGK1 bound to TZ for each configuration. Darker shades of green indicate a greater percent of a TZ-bound configuration. We varied the font between black and white for clarity against light and dark background colors.

In the absence of TZ, reaction of substrates produces products (Fig. 4*A*). There was a strong preference for PGK1 to bind 1,3-BPG first and then ADP. Following phosphotransfer, there was a preference for ATP and then 3-PG to dissociate.

In the presence of 50 nM TZ, a new cycle of activity appeared, revealing how TZ could increase ATP production (Fig. 4*B*). After phosphotransfer, ATP releases from E⋅ATP⋅PG forming E⋅PG. In the presence of 50 nM TZ, the strong association of TZ formed E⋅TZ⋅PG, and E⋅PG decreases. This changes the configuration for 3-PG release from E⋅PG to E⋅TZ⋅PG and the release parameter for 3-PG from *c-* to *b-*_._ 3-PG releases more readily from E⋅TZ⋅PG than from E⋅PG because *b-* is greater than *c-*. Then, the clockwise progression of the TZ-bound enzyme around the new cycle ultimately increases E⋅ADP⋅BPG, leading to another round of enzymatic phosphotransfer. This new cycle circumvents the slow release of 3-PG from E⋅PG at the end of the PGK1 reaction.

At 25 μM, TZ acts as a true competitive inhibitor (Fig. 4*C*). With TZ binding 93% of the PGK1, the percentage change for each non-TZ-bound configuration decreases, and the net flux for each interaction approaches zero. Thus, TZ binding inhibits the reaction from taking place. Although the association parameter for TZ is not known, the *Kd* for TZ is smaller than for ADP, and TZ’s association parameter is modeled at 562 times larger than that of ADP, which allows low concentrations of TZ to compete with higher ADP concentrations. This can be seen in Tables 2 and 3 and Fig. 4*C*, where the E⋅TZ⋅BPG configuration contains ~91% of the total enzyme caused by TZ’s relatively high association rate and concentration.

To gain further understanding of how the kinetic properties of PGK1 allow the unusual biphasic behavior from a competitive inhibitor, we assumed that the release of 3-PG from E⋅PG is slower, governed by the *c-* parameter, than the other dissociation rates (*a-*, *b-*, and *d-*). This step is highlighted in Fig. 4*D*. Adding TZ provides an alternate route for 3-PG release, 3-PG can dissociate from E⋅TZ⋅PG, and importantly, the *b-* parameter is faster than *c-*. We also explored the effect of varying the ATP release parameters (*a-* and *d-*) on ATP production (*SI Appendix*, Figs. S1 and S2).

**Fig. 5.**
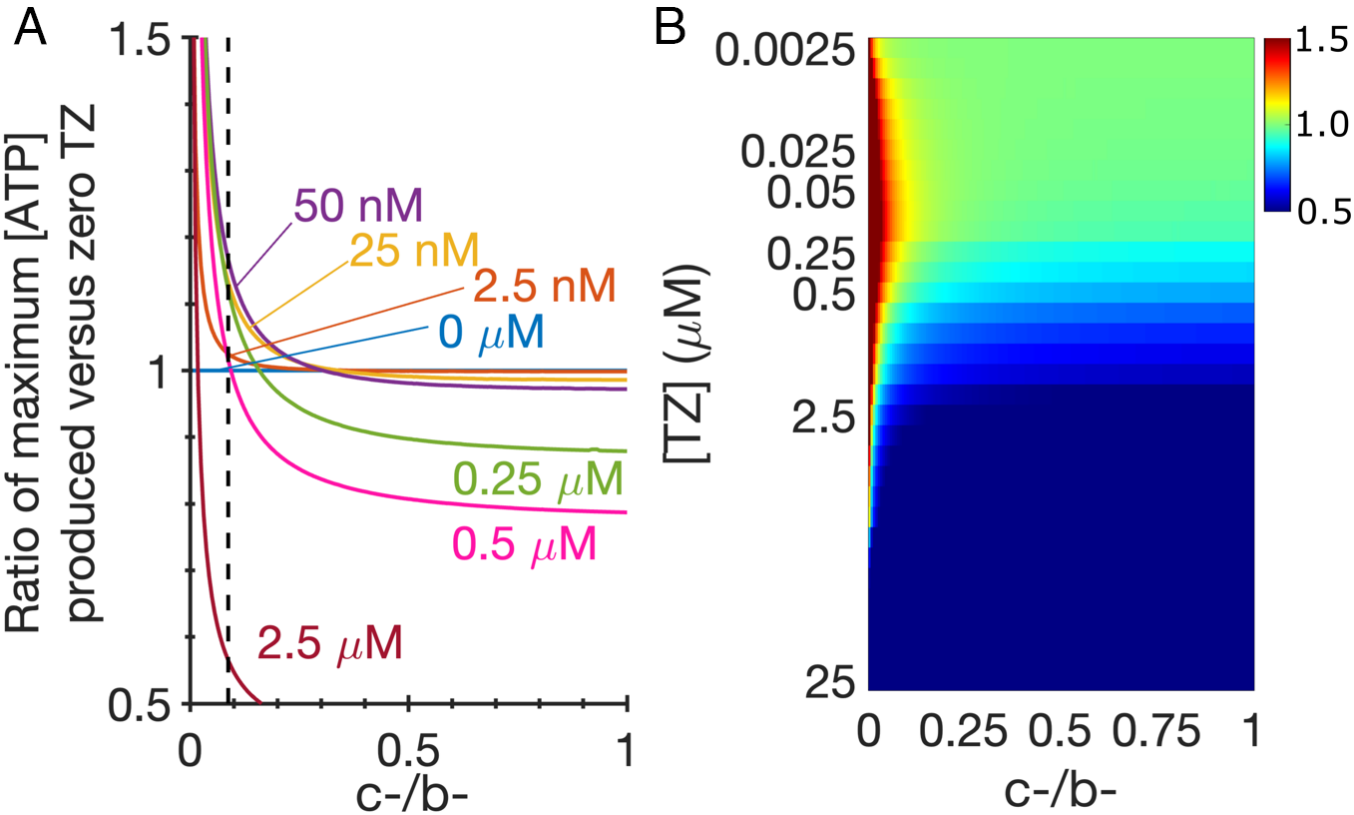
The ratio of *c-*/*b-* determines the effect of TZ on ATP production. For each panel, we fix *b-* = 160 s^−1^ (Table 1) and vary *c-*. (*A*) The black-dashed line is *c-/b-* from Table 1. The colored curves correspond to TZ concentrations. As the *c-/b-* ratio increases, the ratio of ATP produced in the presence of TZ versus no TZ decreases. As *c-/b-* approaches one, the inhibitory behavior of TZ becomes apparent because the release of 3- PG can occur via *c-* and the competitive binding of TZ works to only impede ATP production. (*B*) A heat map shows several TZ concentrations with an associated *c-/b-* value. A small *c-/b-* ratio shows a stimulatory effect (red/yellow) and larger *c-/b-* values show either no effect (green) or inhibition (blue).

To explore how the relative sizes of *c-* and *b-* influenced ATP production, we repeated simulations while varying *c-*, fixing b-, and determined the ATP production rate at each TZ concentration relative to that without TZ (Fig. 5*A*). When the *c-/b-* ratio is close to zero, all TZ concentrations stimulate ATP production. However, as the *c-/b-* ratio increases, intermediate TZ concentrations no longer stimulate. As *c-/b-* approaches one, larger TZ concentrations decrease ATP production, and intermediate concentrations no longer provide the benefit of stimulating PGK1 activity, since PG can release from E⋅PG. The vertical, black-dashed line in Fig. 5*A* is the value of *c-/b-* from Table 1. In Fig. 5*B*, we show the ratio of ATP production with TZ versus with no TZ as a heat map. When TZ had no effect on ATP production, the ratio is one (green). Low TZ concentrations increased (red), and high concentrations decreased (blue) ATP production. The magnitude of the response and the effect of TZ concentration depend on the *c-/b-* ratio.

## Discussion

This model explains two paradoxes about the interaction between TZ and PGK1. First, how can a competitive inhibitor increase the activity of an enzyme? We found that TZ introduces an alternative pathway that bypasses slow release of 3-PG from E⋅PG by generating E⋅TZ⋅PG. The TZ-bound enzyme facilitated 3-PG product release, cycled the enzyme to reload ADP and 1,3-BPG, and accelerated another round of enzymatic phosphotransfer. Second, what might be the basis for the biphasic relationship between TZ concentration and PGK1 activity? Low TZ concentrations can stimulate PGK1 activity as described above. But after entering the new bypass cycle, high TZ concentrations can prevent PGK1 from leaving the cycle, behaving as a competitive inhibitor.

The mechanism by which a competitive inhibitor at low concentrations (TZ) can accelerate enzymatic (PGK1) activity appears robust. The TZ⋅PGK1 bypass cycle would be eliminated if the ratio of (E⋅ATP⋅PG → E⋅PG) / (E⋅ATP⋅PG → E⋅ATP), i.e., *d-/b-*, became zero. This would force E⋅ATP⋅PG to release PG first leaving no pocket for TZ to bind on E⋅ATP, and therefore there would be no entry into the bypass cycle. Although measures of these dissociation rates are not available, the reverse of these two transitions occurs, and the *Kd* of those steps are within 10% of each other (17). Thus, it is very unlikely that the *d-/b-* ratio would be zero. If the ratio of (E⋅PG → E) / (E⋅TZ⋅PG → E⋅TZ), i.e., *c-/b-*, increases and becomes greater than 1, the TZ⋅PGK1 bypass cycle would no longer provide any benefit. However, the affinity of TZ is 10 to 100 times greater than that of ADP and ATP, which shuttles E⋅PG → E⋅TZ⋅PG setting up 3-PG release from that configuration; we described how changes in *c-/b-* impact 3-PG release above. Consequently, while we have made several approximations to rate parameters in our simulations, we conjecture that any choice of non-zero parameters with a sufficiently small *c-/b-* ratio can exhibit biphasic dose dependence.

The approach and model have advantages and limitations. An advantage is that the configurations in the model were assigned based on known interaction sites between the enzyme, substrates, products, and TZ (5, 6). In addition, we used previously published kinetic measurements to determine reaction rates (6). A limitation is that the association and dissociation parameters of ATP and 3-PG were unavailable, and we therefore approximated that they were the same as those for ADP and 1,3-BPG. The phosphotransfer parameters *k_±_* were also arbitrarily assigned a value. Modifying these parameters would change the quantification of PGK1 activity. The quantification of PGK1 activity would also vary with substrate concentrations, and here we used a single set of concentrations. However, due to the model’s robustness as outlined in the preceding paragraph, the qualitative understanding of the bypass cycle would remain valid.

After developing and analyzing this mechanistic model, we learned that a somewhat similar model was reported more than six decades ago by Theorell and McKee (20). They studied liver alcohol dehydrogenase, which converts an alcohol and NAD^+^ to acetaldehyde and NADH. They found that imidazole generates a ternary complex of enzyme⋅imidazole⋅NADH. Their kinetic measurements indicate that the ternary complex releases NADH more readily than does enzyme⋅NADH, hence it creates a potential bypass pathway. Low imidazole concentrations accelerate product release, whereas high concentrations competitively inhibit enzymatic activity.

There are other models by which inhibitors can paradoxically stimulate enzyme activity. As an example, ATP-competitive inhibitors of rapidly accelerated fibrosarcoma (RAF) kinase can increase enzyme activity (21–23). The inhibitor binds to one member of a wild-type RAF dimer which transactivates the other drug-free member of the dimer thereby increasing its kinase activity. At high concentrations, the inhibitors reduce kinase activity. Importantly for cancer treatment, mutant RAF has minimal transactivation, and thus, competitive inhibitors decrease kinase activity. An example with a different mechanism is a substrate-derived inhibitor of heat shock enzyme DegP that stimulated DegP activity at low concentrations (24). The inhibitor interacted allosterically with DegP resulting in increased affinity of unoccupied sites, ultimately increasing enzymatic activity. High concentrations of the allosteric inhibitor diminished enzymatic activity by inhibiting all unoccupied sites.

Beyond PGK1, these findings may have implications for agents that interact with the active site of other enzymes. PGK1 has two substrates and two products, which suggests that comparable bypass models might exist for multiple other metabolic enzymes with two substrates and two products. Enzymes are the target for many existing and potential medicines; in many cases, the goal has been to inhibit activity, often by targeting the active site of the enzyme. Understanding how an agent might either inhibit or stimulate an enzyme depending on concentration might explain confusing outcomes and provide opportunities for other discoveries.

This model also has practical implications. First, the model supports the conclusion that TZ enhancement of PGK1 activity and the biphasic dose–response relationship are inherent properties of the interaction between the enzyme and TZ. Second, by providing a plausible mechanism for the biphasic TZ dose–response observed in cells and animals, it highlights that in vivo dosing will require careful attention to beneficially enhance energy metabolism. Thus, clinical testing of TZ as an agent that may slow or prevent neurodegeneration may require knowledge of the relationship between the blood TZ concentration and the position on the TZ dose–response. Pilot data suggest that measurements of the effect of TZ on ATP levels in blood or the brain might suffice (9). Third, as a potential therapeutic for neurodegenerative disease, TZ has the limitation that it also inhibits α_l_-adrenergic receptors and can thus predispose to orthostatic hypotension (25). Development of novel agents that stimulate PGK1 activity but lack α_l_-adrenergic receptor antagonism will benefit from the understanding provided by this model. Thus, this work may provide a foundation for additional experimentation and development.

## Materials and Methods

PGK1 activity was measured using a coupled reaction with GAPDH (5). The assay buffer contained 50 mM Na_2_HPO_4_, 100 mM triethanolamine, 5 mM MgSO_4_, and 0.2 mM ethylenediaminetetraacetic acid. The solution was titrated to pH 8.6 with concentrated HCl. Substrates were added to a concentration of 1 mM NAD and 1 mM glyceraldehyde 3-phosphate (GAP). The solution was incubated for 5 min after addition of 5 U/mL of GAPDH and 50 ng/mL of PGK1. Substrates and chemicals were from Sigma-Aldrich, Missouri, USA, including GAP (g5251) and GAPDH (g2267). Human PGK1 was from Abcam (ab289789) Cambridge, UK. The reaction was initiated with 1 mM ADP, and the reaction was monitored by measuring the absorbance of NADH at 339 nm using a CARY 3500 spectrophotometer (Agilent). TZ (10 µM) or vehicle (water) was added to each cuvette with a 1/1,000 dilution. We fit PGK1 activity data to the Michaelis–Menten equation (26, 27).

## Supplementary Material

Appendix 01 (PDF)

## Data Availability

All study data are included in the article and/or *SI Appendix*.
