# Supplementary material for: A model for stimulation of enzyme activity by a competitive inhibitor based on the interaction of terazosin and phosphoglycerate kinase 1
Source: Proc Natl Acad Sci U S A. 2024 Feb 20;121(9):e2318956121. doi: 10.1073/pnas.2318956121 (PMC10907273; doi:10.1073/pnas.2318956121)
Supplement: Supplementary file 1 — Appendix 01 (PDF) [file pnas.2318956121.sapp.pdf]

PNAS

Supporting Information for

A model for stimulation of enzyme activity by a competitive inhibitor based on the interaction of terazosin and phosphoglycerate kinase 1

Mitchell J Riley[1,2*], Colleen C Mitchell[1*], Sarah E Ernst[2,3], Eric B Taylor[4], Michael J Welsh[2,3,4]

Address communications to Colleen Mitchell or Michael Welsh.
Email: colleen-mitchell@uiowa.edu or michael-welsh@uiowa.edu

This PDF file includes:

Supporting text
Figures S1 to S2
Appendix

Supporting Information Text

Below, Figure S1 and Figure S2 show how ATP production is affected by varying the release parameters *a*- and *d*-.

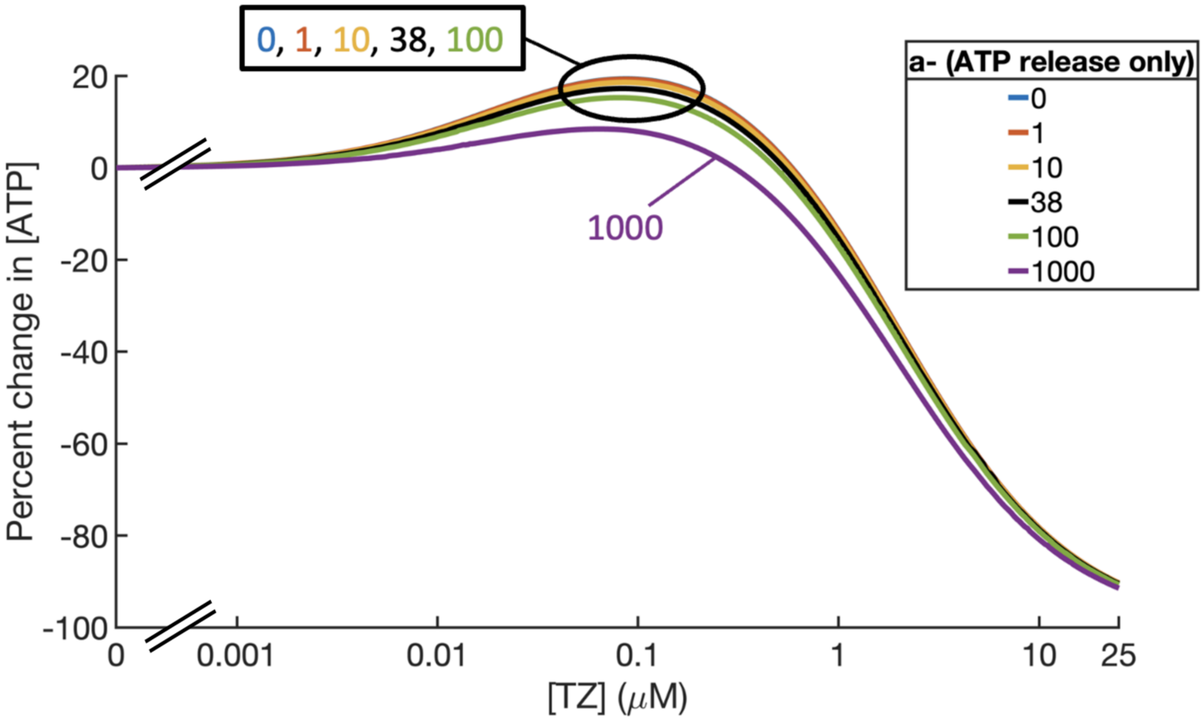

Fig. S1. Effect of *a-* for ATP release from E·ATP on the production of ATP. A simulation showing the percent change in ATP production at varying TZ concentrations at 1 minute using parameters from Table 2 (except *a-*) and initial conditions outlined in the methods.

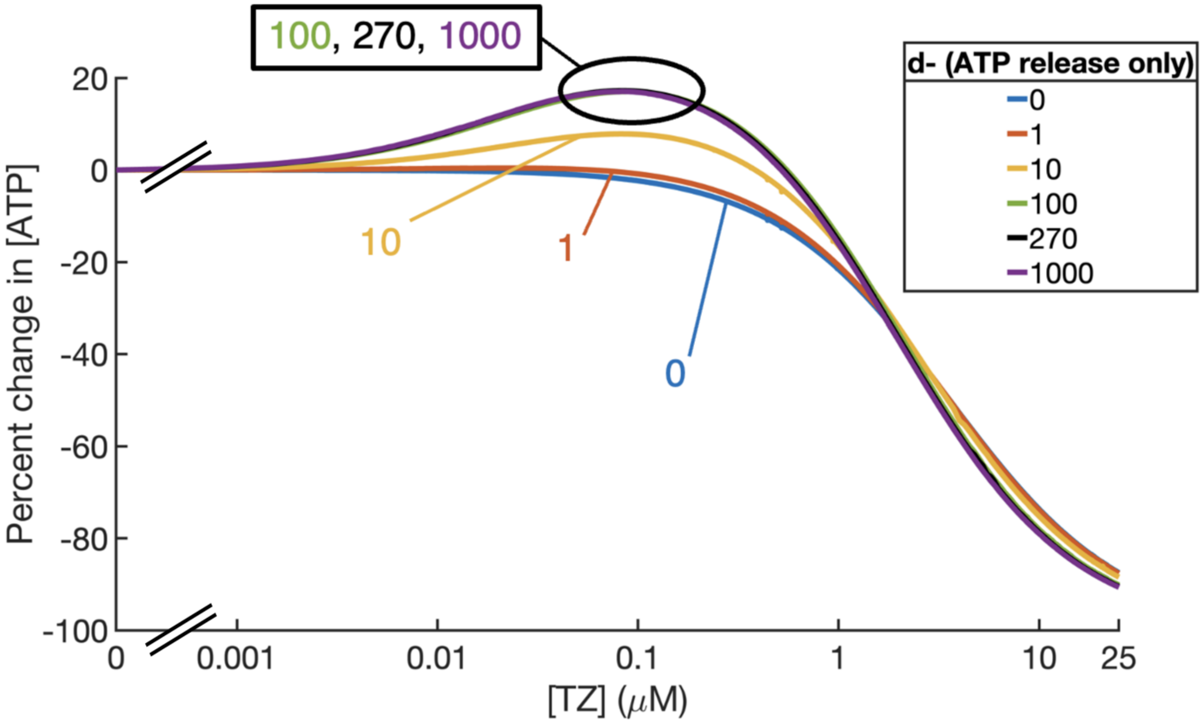

Fig. S2. Effect of *d-* for ATP release from E·ATP·PG on the production of ATP. A simulation showing the percent change in ATP production at varying TZ concentrations at one minute using parameters from Table 2 (except *d-*) and initial conditions outlined in the methods.

Appendix

$$(E)\ \dot{x_1} = -a_+x_1x_2 - c_+x_1x_3 + a_-x_4 + c_-x_5 - a_+x_1x_9$$

$$-c_+x_1x_8 + a_-x_{10} + c_-x_{11} - \eta a_+x_1z_1 + a_-z_2$$

$$(ADP)\ \dot{x_2} = -a_+x_1x_2 - d_+x_2x_5 + a_-x_4 + d_-x_6$$

$$(1{,}3 - BPG)\ \dot{x_3} = 0$$

$$(E \cdot ADP)\ \dot{x_4} = -a_-x_4 - b_+x_4x_3 + a_+x_1x_2 + b_-x_6$$

$$(E \cdot BPG)\ \dot{x_5} = -c_-x_5 - d_+x_5x_2 + c_+x_1x_3 + d_-x_6 - \eta d_+z_1x_5 + d_-z_3$$

$$(E \cdot ADP \cdot BPG)\ \dot{x_6} = -b_-x_6 - d_-x_6 + b_+x_4x_3 + d_+x_5x_2 - k_+x_6 + k_-x_7$$

$$(E \cdot ATP \cdot PG)\ \dot{x_7} = -b_-x_7 - d_-x_7 + b_+x_8x_{10} + d_+x_9x_{11} - k_-x_7 + k_+x_6$$

$$(3 - PG)\ \dot{x_8} = -b_+x_8x_{10} - c_+x_1x_8 + b_-x_7 + c_-x_{11} - b_+x_8z_2 + b_-z_4$$

$$(ATP)\ \dot{x_9} = -a_+x_1x_9 - d_+x_9x_{11} + a_-x_{10} + d_-x_7$$

$$(E \cdot ATP)\ \dot{x_{10}} = -a_-x_{10} - b_+x_8x_{10} + a_+x_1x_9 + b_-x_7$$

$$(E \cdot PG)\ \dot{x_{11}} = -c_-x_{11} - d_+x_9x_{11} + c_+x_1x_8 + d_-x_7 - \eta d_+z_1x_{11} + d_-z_4$$

$$(TZ)\ \dot{z_1} = -\eta d_+z_1x_5 - \eta d_+z_1x_{11} - \eta a_+x_1z_1 + a_-z_2 + d_-z_3 + d_-z_4$$

$$(E \cdot TZ)\ \dot{z_2} = -b_+x_3z_2 - b_+z_2x_8 - a_-z_2 + b_-z_4 + \eta a_+z_1x_1$$

$$(E \cdot TZ \cdot BPG)\ \dot{z_3} = -d_-z_3 - b_-z_3 + \eta d_+z_1x_5 + b_+x_3z_2$$

$$(E \cdot TZ \cdot PG)\ \dot{z_4} = -d_-z_4 - b_-z_4 + \eta d_+z_1x_{11} + b_+x_8z_2$$
